# Optimization of dose distribution with multi-leaf collimator using field-in-field technique for parallel opposing tangential beams of breast cancers

**DOI:** 10.4103/0971-6203.41194

**Published:** 2008

**Authors:** K. Krishna Murthy, S. S. Sivakumar, C. A. Davis, R. Ravichandran, Kamal El Ghamrawy

**Affiliations:** Department of Radiotherapy, National Oncology Center, The Royal Hospital, Muscat, Sultanate of Oman

**Keywords:** 3D-CRT, dose volume histogram, homogeneity index, planned target volume

## Abstract

3 Dimensional Conformal Radiotherapy (3D-CRT) planning software helps in displaying the 3D dose distribution at different levels in the planned target volume (PTV). Physical or dynamic wedges are commonly applied to obtain homogeneous dose distribution in the PTV. Despite all these planning efforts, there are about 10% increased dose hot spots encountered in final plans. To overcome the effect of formation of hot spots, a manual forward planning method has been used. In this method, two more beams with multi-leaf collimator (MLC) of different weights are added in addition to medial and lateral wedged tangent beams. Fifteen patient treatment plans were taken up to check and compare the validity of using additional MLC fields to achieve better homogeneity in dose distributions. The resultant dose distributions with and without presence of MLC were compared objectively. The dose volume histogram (DVH) of each plan for the PTV was evaluated. The 3D dose distributions and homogeneity index (HI) values were compared. The 3D dose maximum values were reduced by 4% to 7%, and hot spots assumed point size. Optimizations of 3D-CRT plans with MLC fields improved the homogeneity and conformability of dose distribution in the PTV. This paper outlines a method of obtaining optimal 3D dose distribution within the PTV in the 3D-CRT planning of breast cases.

## Introduction

The issue of radiation dose delivery to the chest wall after total mastectomy, as well as the treatment technique of conservative breast, remains complex. This is due to the irregularity in contour at the chest wall level and also varying thickness of lungs underneath. Randomized trials have indicated that radiation can improve the overall survival of patients, with a 25% to 30% 10-year risk of loco-regional relapse (LRR), treated with mastectomy and systemic treatment. [1–5]

The CT images with three-dimensional (3D) planning software help in displaying the dose variations at different levels below skin. Physical or dynamic wedges are commonly applied to correct for entrance obliquities. In conventional 3D-CRT for breast cancer, most commonly, either physical or dynamic wedges are used in two tangential fields to achieve optimal three-dimensional dose distribution within the minimal degree of dose inhomogeneity through forward treatment planning. The possibilities and the limits of commonly used techniques for irradiation of breast with two tangential fields in the supine position have been discussed in recent years.[6–8] The early and late complications of radiations are directly related to patient anatomy, total dose delivered, fractionation scheme, and radiation treatment technique. Several institutions have reported the use of different techniques to improve dose distribution within the breast.[9–10]

In conventional method of planning, despite all planning efforts, there are about 10% increased dose hot spots encountered in final plans. In intensity-modulated radiotherapy (IMRT), with inverse planning or forward planning, it is possible to overcome this problem.[11] To overcome the effect of formation of hot spots, we have tried a manual forward planning method using MLC-optimized field-in-field technique. In this case, two more fields are added with multi-leaf collimator (MLC) of different weights in addition to plain medial and lateral wedged tangent beams. In this article, we have described the Methodology of treatment planning adopted for a typical case and furnished DVH comparison results of 15 cases planned with conventional technique of two parallel opposing tangential fields and MLC-optimized filed-in-field technique.

## Materials and Methods

Computerized 3D radiation treatment planning system (RTPS) Eclipse (version 6.5, Varian Ag, USA) was used for treatment planning. High energy linac Clinac 2300 CD (Varian Ag, USA) having 120-leaf millennium MLC was used for the breast field tangential treatments. Fifteen patient treatment plans were taken up to check and compare the validity of using additional MLC fields to achieve better homogeneity in dose distributions. The CT images of 5-mm thickness at different transverse sections away from midplane were taken to create a 3D image. Initially the 3D-CRT planning was done in the conventional way by using two parallel opposing tangential fields with or without wedges. Then two more subfields with MLC were added, and this field-in-field technique was used to smooth out the hot spots. The MLC positions and beam weightings were optimized by forward planning for reduction of hot spots and achievement of better homogeneous dose distribution.

Initially, medial and lateral tangent asymmetrical half beam block fields were designed, using SSD technique, with gantry angles that create a nondivergent posterior field edge and cover the targeted structure. The dose distribution of two fields was examined after normalization of the plan with a reference point with or without wedges, which gave optimal dose to the target volume. Field weightings were adjusted to achieve maximum possible uniform distribution in the target volume.

**Figure 1:**
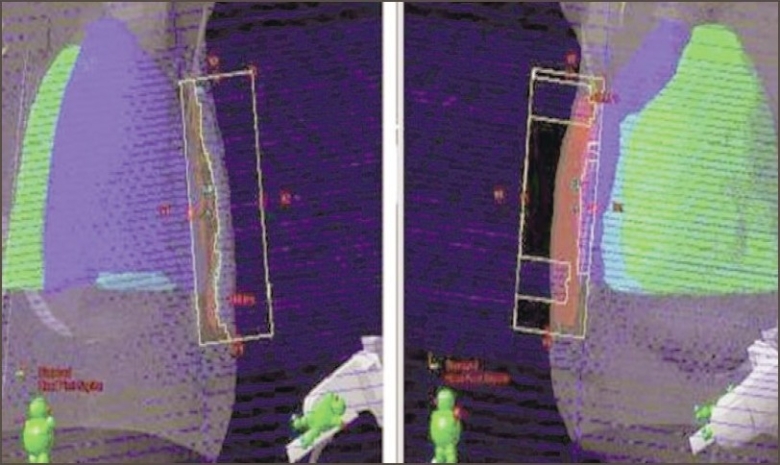
Arrangement of MLC leaves in two tangential open fields. In the BEV of first field, the MLC segments have covered 105% and above of the region of isodose curves. In the BEV of second field, the MLC segments have covered the other hotspots

Then, two more open fields similar to tangential fields were created. The highest hot spot formed at the apex of a breast was projected in the BEV with isodose curves of 105% and above. The region was covered with the segments of MLC from one of the open tangential fields. A small weight was given to this field, and the plan was recalculated. Again the hot spots formed were projected in the BEV, and the region was covered with the segments of MLC from another open tangential field. Again a small weight was given to this field and the plan was recalculated for the distribution of the dose. The arrangement of MLC leaf positions used in two tangential fields is shown in Figure 1. Finally the dose distribution was optimized with the four fields by adjusting the leaf positions and weights of MLC fields. In cases of intact breast, symmetrical fields with SAD technique are used.

The 3D-max, mean, modal, and median dose values of 15 cases planned with conventional two tangential fields and MLC-optimized field-in-field technique were compared using their dose volume histograms. The homogeneity index (HI) for each plan was calculated using the following formula and the mean values were compared. Homogeneity index (HI)=(Dose Max−Dose Min)/Dose Mean in PTV

The significance of HI is that a lesser value of HI indicates greater 3D dose homogeneity in the planned target volume (PTV).

## Results

**Figure 2:**
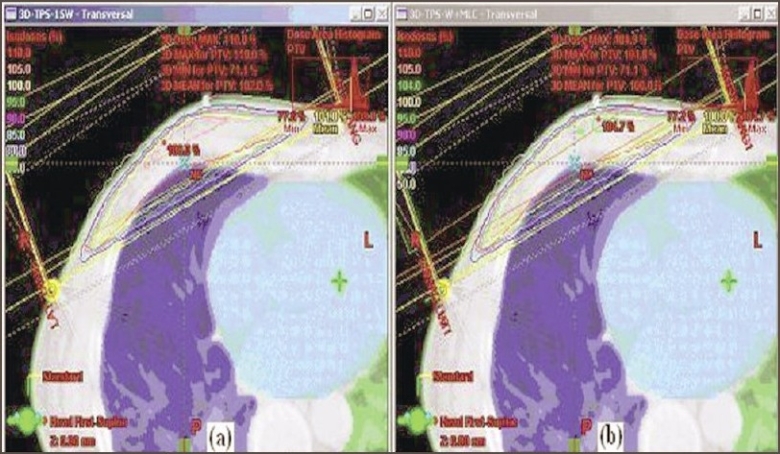
a: Dose distribution with DMax point in a transverse midplane with wedge fields. b: Dose distribution in the same plane with wedge and MLC fields. The 105% and above isodose line hot spots are found removed with the use of MLC fields

**Figure 3:**
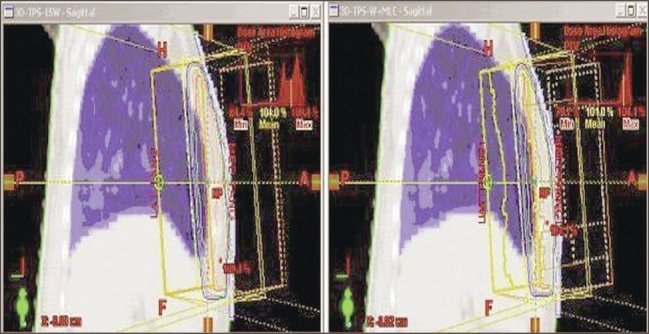
Dose distribution in a sagittal plane with and without MLC fields. The DMax value is found reduced from 110% to <105% with the use of MLC fields

**Figure 4:**
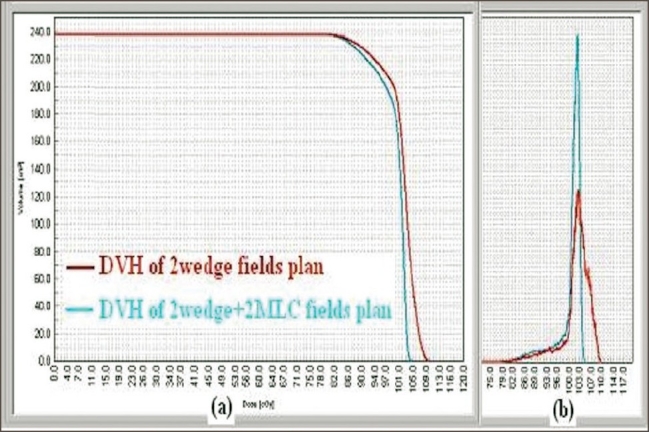
a: Cumulative plan comparison DVH for PTV of two plans. The curve with MLC fields shows that the hot spot is reduced. b: Differential plan comparison DVH for PTV of two plans. The curve with MLC fields shows that the dose coverage of PTV has improved

The dose distributions obtained in a typical plan with and without usage of MLC in transverse midplane and sagittal plane are shown in Figures 2 and 3 respectively. The plan comparison dose volume histograms (DVH) of the case planned are shown in Figure 4. The dose maximum was found reduced from 110% to 105%; and 3D median dose, from 103.1% to 101.8% — showing better distribution and coverage of dose in the PTV with MLC plan than with the simple wedge plan. The dose variation analysis result of 15 breast cases compared with dose volume histogram (DVH) values are shown in Table 1. The mean values of homogeneity index (HI) for the 15 plans with and without MLC were 0.9441 and 0.9762 respectively.

**Table 1:** Dose variation analysis of 15 cases by conventional and multi-leaf collimator-optimized techniques with plan comparison dose volume histogram values

*3D-PTV coverage*	*Range of % dose distribution with 2 primary wedge fields*		*Range of % dose distribution with 2 primary wedge fields and 2 MLC sub fields*		*Range of % dose reduction*	
			
	Actual	Mean	Actual	Mean	Actual	Mean
Max-dose	107-113	111.8±2.5	103-106	106±1.7	4-7	5±0.98
Mean-dose	96-100.5	97.2±2.9	95-99.5	95±2.97	0.2-2.5	2±0.99
Modal-dose	100-105.5	102.3±1.8	99.5-103	100.9±1.5	0.2-2.5	2±0.98
Median-dose	97-103.28	100.8±2.1	96-101.5	99.2±1.79	0.3-3	2±0.88

## Discussion

The cumulative and differential forms of plan comparison histograms shown in Figure 4 clearly indicate that the 3D dose distribution is more confined to the PTV with the MLC-optimized field-in-field technique. The DVH curves show that the hot spot is reduced at the compromise of the PTV dose coverage. This is because the MLC segments not only reduce the hot spots but also reduce the mean dose to the PTV. But still the PTV modal and median doses are close to 100%, and a definite gain is achieved regarding dose homogeneity within the PTV coverage by using this technique. Table 1 gives the difference of the dose distributions in the planned target volume (PTV) with conventional and MLC-optimized field-in-field techniques evaluated by score functions. The technique of MLC field reduced the 3D maximum dose by 4% to 7% (from 113% to 105%). In most of the plans, the dose maximum hot spots have become point doses, and the isodose levels in the range of 95% to 100% cover maximum PTV. The median maximum dose delivered reduced, varying from 0.3% to 3%. In this technique, less than 0%-2% of the treated volume received more than 105% of the prescribed dose compared to 7%-20% of the treated volume in the wedge plans without MLC fields. The technique clearly shows that the dose variation in the PTV becomes less than 5% of the prescribed dose. Hence it is expected that with this technique, the morbidity of skin reactions may be reduced.

The homogeneity index (HI) values also confirmed that the 3D dose distributions were more homogeneous within the PTV with the MLC fields, with a mean value of 0.9441 compared to 0.9762 for the conventional two tangential fields. Though the maximum, minimum, and mean doses were reduced in the 3D PTV with MLC fields, the percentage of reduction in maximum dose was more compared to that in minimum and mean doses. Therefore, the dose homogeneity was achieved with the MLC-optimized field-in-field technique. The results obtained in our study are similar and comparable with the earlier studies of 3D-CRT forward planning of IMRT for breast cases, which have indicated that the dose distribution with MLC treatment improves compared to that with conventional wedge plans[12–15]

## Conclusion

Optimizations of 3D-CRT plans with MLC fields have improved the homogeneity and conformability of dose distribution in the target volume. The homogeneity index (HI) values also confirmed the better 3D dose homogeneity in the PTV with the MLC-optimized field-in-field technique. With this method, the maximum dose value and its coverage area in the PTV reduced considerably. The method helps to understand the role of MLC in overcoming 3D dose inhomogeneity in the 3D-CRT planning of breast cases.
